# Molecular Cloning and Characterisation of Farnesyl Pyrophosphate Synthase from *Tripterygium wilfordii*

**DOI:** 10.1371/journal.pone.0125415

**Published:** 2015-05-04

**Authors:** Yu-Jun Zhao, Xin Chen, Meng Zhang, Ping Su, Yu-Jia Liu, Yu-Ru Tong, Xiu-Juan Wang, Lu-Qi Huang, Wei Gao

**Affiliations:** 1 Capital Medical University School of Traditional Chinese Medicine, Capital Medical University, Beijing, People’s Republic of China; 2 National Resource Center for Chinese Materia Medica, China Academy of Chinese Medical Sciences, Beijing, People’s Republic of China; Texas A&M University, UNITED STATES

## Abstract

Farnesylpyrophosphate synthase (FPS) catalyzes the biosynthesis of farnesyl pyrophosphate (FPP), which is an important precursor of sesquiterpenoids such as artemisinin and wilfordine. In the present study, we report the molecular cloning and characterization of two full-length cDNAs encoding FPSs from *Tripterygium wilfordii (TwFPSs)*. *TwFPSs* maintained their capability to synthesise FPP *in vitro* when purified as recombinant proteins from *E*. *coli*. Consistent with the endogenous role of FPS in FPP biosynthesis, *TwFPSs* were highly expressed in *T*. *wilfordii* roots, and were up-regulated upon methyl jasmonate (MeJA) treatment. The global gene expression profiles suggested that the *TwFPSs* might play an important regulatory role interpenoid biosynthesis in *T*. *wilfordii*, laying the groundwork for the future study of the synthetic biology of natural terpene products.

## Introduction

**Fig 1:**
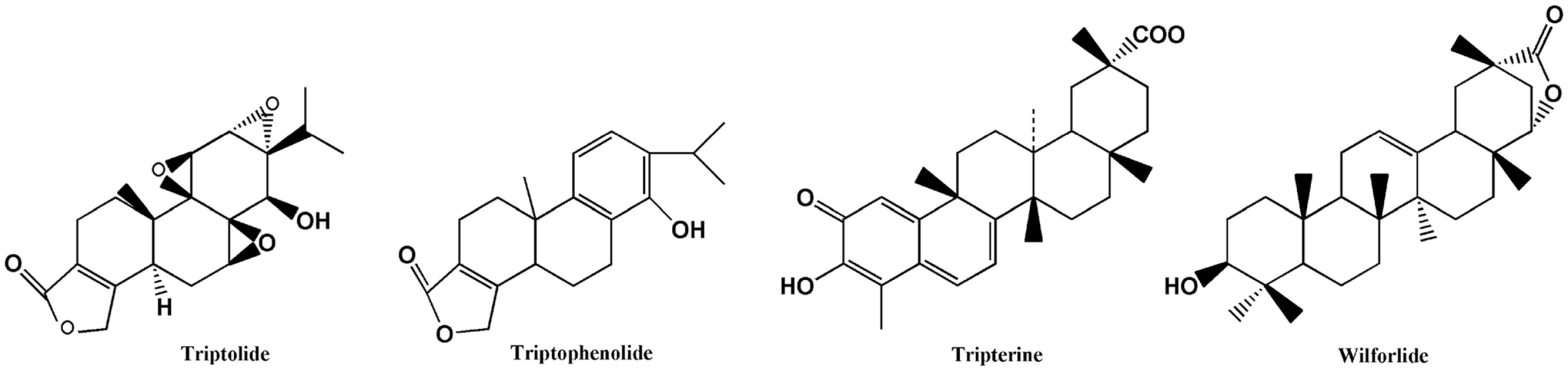
The main medicinal active substances of *Tripterygium wilfordii*.

*Tripterygium wilfordii* is a traditional Chinese medicinal plant used to treat inflammatory diseases because of its analgesic and anti-microbial properties [1]. This plant has been widely used in the treatment of immune and tumour diseases [2–4]. Terpenoids are the primary active substances (Fig 1) of *T*.*wilfordii* and include sesquiterpene, diterpenoids andtriterpenoids. Triptolide [5], which has been recognised as one of the primary active constituents of *T*. *wilfordii*, is a class of 3 epoxygroups and an α,β-unsaturated five-membered lactone ringand is also a unique abietane diterpene. *T*. *wilfordii* has attracted much attention due to its architecture and significant activities. Tripterine [6], which was the first monomer isolated from *T*. *wilfordii* has important biological activity.

FPS belongs to the family of short-chain prenyltransferases [7]. FPS catalyzes the head-to-tail condensation reaction of dimethylallyl pyrophosphate (DMAPP) with two molecules of isopentenyl pyrophosphate (IPP) to form FPP [8–9], which is the precursor of all sesquiterpenes [10], such as artemisinin and wilfordine.FPS provides substrate FPP to squalene synthase and sesquiterpene synthase. Squalene synthase brings the synthesis into steroids and saponins, which involved in cell membrane systems building; sesquiterpene synthase put the synthesis on cyclic sesquiterpene compounds [11]. Upon treatment with elicitors, FPP, which is normally employed in sterol biosynthesis, is diverted from the sterol pathway as large amounts of antibiotic sesquiterpenesare produced [12–14]. This diversion is accomplished by the induction of sesquiterpene cyclases and coeval suppression of squalene synthesis, the initial committed step of phytosterol production [13–14].

Thus far, FPS has been isolated from sorghum [15], corn [16], sandalwood [17] and 41 other species of plants. Recently, FPS has been isolated from *Panax ginseng* [18], *Salvia miltiorrhiza*, *Acanthopanax senticosus*, Artemisia annua [19], *Ginkgo biloba* [20] and other medicinal plants. International and domestic academics have studied the gene expression patterns of farnesyl pyrophosphate synthase in plants and found that this gene has tissue-specific expression with increased isoprenoid derivative contents. Gene expression studies have demonstrated that gene expression is related to the isoprene content in plants.

The effective components in *T*. *wilfordii* are difficult to obtain by traditional chemical methods. In addition, the products of bioactive compounds cannot be synthesized through microbial methods. The current information regarding terpenoid biosynthesis genes is limited, and studies regarding key enzymes in *T*. *wilfordii* are few. Based on the above issues, we present the cloning and characterisation of full-length FPS cDNAs of *T*. *wilfordii* (*TwFPSs*) for the first time.

## Materials and Methods

### Plant materials

Fresh leaves and stems of *T*. *wilfordii* were sheared, cleaned and disinfected. Then, the leaves were cut into 1.0 cm×1.0 cm pieces, and the stems were cut into 1.0 cm lengths after rinsing with sterile water. These tissues were cultivated in MS agar medium containing 2,4-D hormone at 25°C in dark. After two weeks, calluses began to grow at the explant slits. The calluses which had a white lustre, were soft and grew well, were cultured in MS agar medium containing 0.5 mg/L of 2,4-D, 0.1 mg/L of KT, and 0.5 mg/L of IBA at 25°C in the dark.

The calluses were cultured in MS agar medium containing 0.2 mg/L of IAA, 0.5 mg/L of KT, and 1.5 mg/L of 6-BA at 25°C under 16 h light/8 h dark conditions. The aseptic seedlings were harvested after subculturing once a month.

After 3 subcultures, we chose the calluses that grew well and that had a loose texture and clipped these calluses into small pieces with tweezers. These calluses were cultured in MS medium containing 0.5 mg/L of 2,4-D, 0.1 mg/L of KT, 0.5 mg/L of IBA, and cell suspensions of 2.0 g/40 mL in the dark at 25°C with rotary shaking at 120 rpm.

### Elicitor treatment

*T*. *wilfordii* cell suspensions displaying good growth were chosen as the experimental material. After 12 days, 1 mM [21] methyl jasmonate (MeJA) [22–23] was added to the treatment groups, and DMSO was added to the control groups. After treatment, the cell suspensions were harvested in liquid nitrogen at 0, 4, 12, 24 and 48 hand stored at -80°C.

### Cloning of *TwFPS1* and *TwFPS2* from *T*. *wilfordii*

Total RNA was extracted from *T*. *wilfordii* cell suspensions using the cetyltrimethylammonium bromide (CTAB) [24] method. Genome or protein contamination was eliminated using DNase I and an RNA purification kit. A PrimeScript 1st Strand cDNA Synthesis Kit was used to obtain the cDNA. A SMART RACE cDNA Amplification Kit was used to transcribe the first-strand cDNA for 3′- and 5′-RACE. The 3′- and 5′-RACE products were subcloned into the pMD19-T plasmid. The cloning vector was amplified in *E*. *coli* DH5α cells. After sequencing and alignment, the full-length predicted cDNAs for *TwFPS1* and *TwFPS2* could be obtained. 3′-RACE Primer: 5′-TGCCTTGCTCGGATGGGCTTCG-3′ (*TwFPS1*), 5′-GGATGATTACCTGGACTGTTTTGGGG-3′ (*TwFPS2*). 5′-RACE primer: 5′-TGGTTGACCCCGCCGAGTAACAGAT-3′ (*TwFPS1*), 5′-TGAACAATGCGGCGGTGGAGTGAC-3′ (*TwFPS2*). New primers were designed to confirm the open reading frame (ORF) of *T*. *wilfordii*. *TwFPS1*: 5′-ACATGGGGATCGGCAGCCATAC-3′ (forward) and 5′-TCAGAAGCTACGGCAGAATCTAATGGAG-3′ (reverse). *TwFPS2*: 5′-TCTCTGTGTCTCCGCAAA-3′ (forward) and 5′-GAGTAACCATAAGCAGCAGAC-3′ (reverse).

### Sequence and phylogenetic analyses

The nucleotide and protein sequences were compared using NCBI (http://www.ncbi.NLM.NIH.gov). The sequences were translated into amino acidsusing DNAMAN software. The ORF was searched using ORF Finder (www.ncbi.NLM.NIH.gov/Gorf/Gorf.html). The theoretical isoelectric point (pI) and molecular weight (Mw) were determined using the Compute pI/Mw tool (http://Web.ExPASy.org/compute_pi/). Multiple sequence alignments were performed using DNAMAN and ClustalW software. Phylogenetic analysis was performed using MEGA5.1 software to build evolutionary trees.

### Expression of *TwFPS1* and*TwFPS2* in *E*. *coli* and purification of recombinant protein

Based on the cloning vector, the primers for recombination were designed; an NcoI site and a HindIII site were introduced at the start and stop codons, respectively. *TwFPS1*: 5′-GAGGAGCCATGGCTATGAGCGACACCAAGTCCAAG-3′ (forward) and 5′-GAGGAGAAGCTTCTACTTCTCTCGCTTGTATAT-3′ (reverse). The primers for recombination were designed; a KpnI site and an EcoRI site were introduced at the start and stop codons, respectively. *TwFPS2*: 5′-GAGGAGGGTACCATGGCGGATCTCAAGTCAACG-3′ (forward) and 5′-GAGGAGGAATTCCTACTTCTGTCTCTTGTATATC-3′ (reverse). The fragments were cloned into the expression vector pET-32a(+). *E*. *coli* BL21 (DE3) cells were used for recombinant plasmid expression and the induction was performed at 16°C for 20 h with the addition of 1 mM isopropyl thiogalactoside (IPTG). After collection, the cells were resuspended in 2 mL lysis buffer A (20 mM Tris—HCl(pH 8.0), 500 mM NaCl, 1 mM PMSF and bacteria protease inhibitor cocktail), lysed by sonication (lysed for 5s, paused for 5s) and centrifuged for 30 min at 13000 g. Thus, the supernatant protein was harvested.

This supernatant was loaded onto a Ni-NTA column and pre-equilibrated by lysis buffer A. The column was washed with 10 column volumes of lysis buffer A containing 20 mM imidazole. The target proteins were eluted by buffer B (20 mM Tris—HCl(pH 8.0) containing 500 mM NaCl and 50 mM imidazole) and buffer C (20 mM Tris—HCl(pH 8.0) containing 150 mM NaCl and 250 mM imidazole). The purified protein was examined by SDS-PAGE gel electrophoresis. The concentration of FPPS protein was determined by the Bradford method [25].

### Assay for enzymatic activity

Scale assays (200μL) for the identification of enzymatic reaction were performed with purified FPPS proteins (200μg) using 100μM IPP and 100μM DMAPP in assay buffer (50 mM Tris—HCl, pH 7.6) containing 5 mM MgCl_2_, 25 mM DTT, and 10% [v/v] glycerol. The assays were incubated at 30°C for 2 hours. To stop the assay and hydrolyse all diphosphate esters, 200μL of solution containing 2 units of potato apyrase and 2 units of calfintestine alkaline phosphatasein 0.2 M Tris—HCl(pH 9.5)was added to all assays, followed by incubation for 8 h at 30°C. After enzymatic hydrolysis, the resulting isopropyl alcohols were extracted into 500μL hexane 3 times. The hexane phase was concentrated by passing N_2_ at the opening of the tube, and then the products were dissolved in 100μL hexane and used for GC—MS measurements.

GC—MS analysis was detected on an Agilent 6890N gas chromatograph (splitless; injector temperature, 250°C) with a 5975imass spectrometer (GC—MS). One microliter of dissolved organic phase was injected. The separation was performed on an HP-5MS column (50 m×0.25 mm×0.5μm) with helium as the carrier gas (flow rate of 1 mL/min) on a temperature gradient from 60°C, at 10°C per min to 90°C (hold 1 min),and then 3°C per min to 220°C (hold 1 min). Mass spectra, 70eV (in EI mode), ion trap heating, 230°C; scan range, 30–500 amu. The products were identified viamass spectrometry profiles, and farnesol could be identified using standard chemicals and retention times. The assay was performed with empty vector as a control.

### Real-time quantitative PCR analysis of *TwFPS1* and*TwFPS2* expression

Total RNA from different tissues (roots, stems, and leaves) and different inductive stages was extracted separately as described above. The primers for real-time quantitative PCR analysis were designed using Primer Premier 5.0 software. *TwFPS1*: 5′-GGGTGTATTTGCGGAGT-3′ (forward) and 5′-CGGCAGAATCTAATGGAG-3′ (reverse); *TwFPS2*: 5′-CAGACCCTCACCTTCCATT-3′ (forward) and 5′-AAGAGTAACCATAAGCAGCAGAC-3′ (reverse). The βtactin gene was used as an endogenous control to normalize expression. The PCR reaction conditions were as follows: an initial incubation at 95°C for 3 min and then cycling at 95°C for 3 s and 60°C for 30 s for 40 cycles. There were three samples in each group and each sample was repeated for three times to insure the credibility of the data. The relative quantification of the *TwFPS* transcript levels was achieved by the 2^-ΔΔCt^ [26] method using ABI 7500 Software v2.0.1 (PE Applied Biosystems).

## Results

### Molecular cloning of the full-length *TwFPS1* and*TwFPS2* cDNAs

RT-PCR was performed with total RNA from *T*. *wilfordii*. *TwFPS* gene fragments were obtained by 3′ rapid amplification of cDNA ends (3′-RACE-PCR) and 5′-RACE-PCR. The full-length cDNA encoding the FPS protein was isolated from *T*. *wilfordii*. The full-length cDNA of *TwFPS1*was 1345bp, with a1029bp ORF, which encodes a 342 amino acid polypeptide, flanked by an 81bp 5′-untranslated region and a 235bp 3′-untranslated region including a 28bp poly(A) tail. The predicted *TwFPS1* protein had a calculated molecular mass of 39.54 kDa and a theoretical pI of 5.59 (GenBank accession number KM058711). The full-length cDNA of *TwFPS2*is1312bp, with a1029bp ORF, which encodes a 342 amino acid polypeptide, flanked by a 68bp 5′-untranslated region and a 215bp 3′-untranslated region including a of 26bp poly (A) tail. The predicted *TwFPS2*protein had a calculated molecular mass of 39.54 kDa and a theoretical pI of 5.28 (GenBank accession number KM058712).

**Fig 2:**
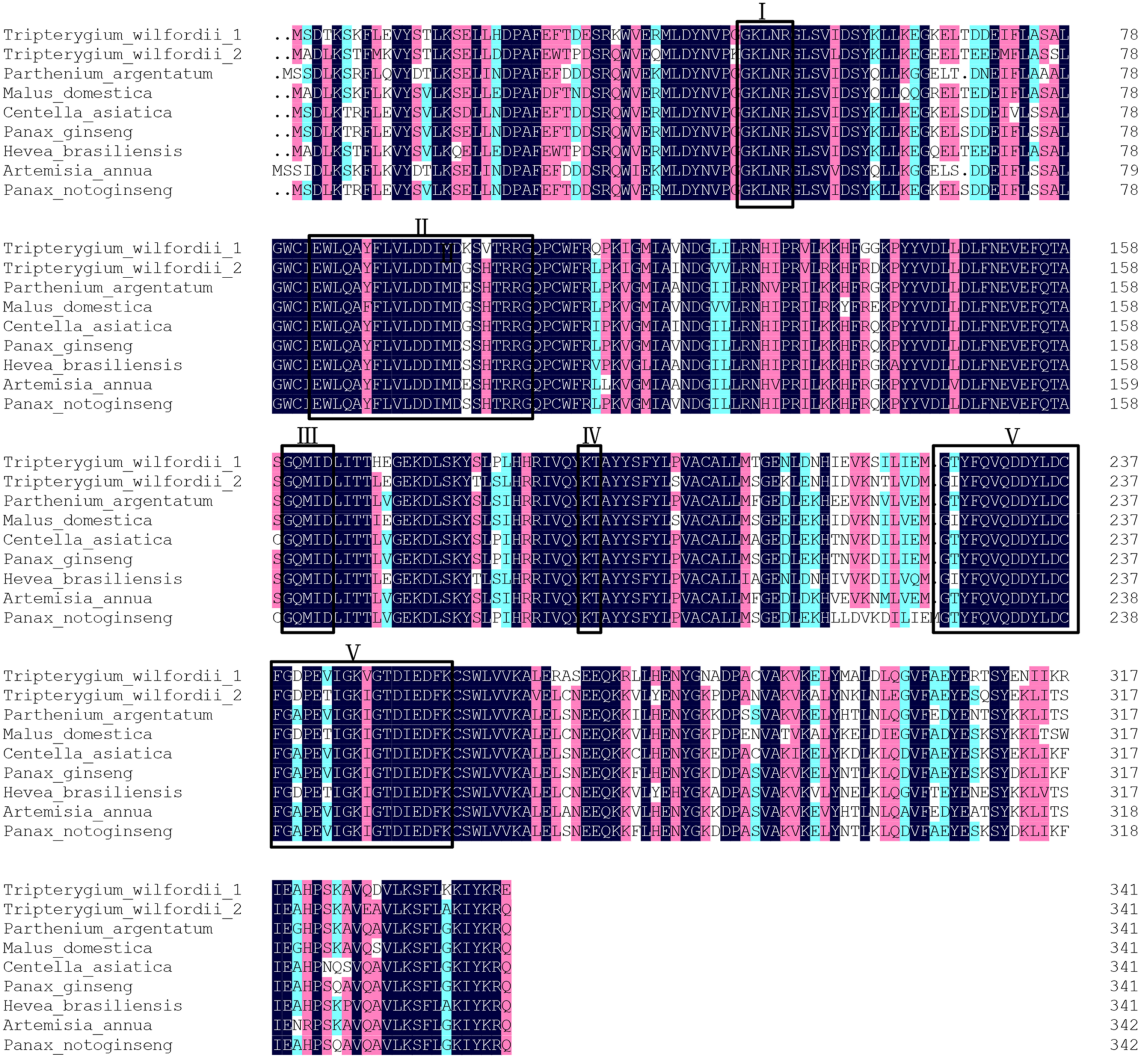
Comparison of the deduced amino acid sequences of*TwFPS1*, *TwFPS2* and related proteins. The five conserved domains of prenyltransferases are boxed and numbered. The highly conserved aspartate-rich motif (DDXX(XX)D) was present in domains II and V.

A BLAST search of the NCBI protein database showed that the deduced amino acid sequence of *TwFPS1* had 77–85% identity to the FPSs from *Mangifera indica*, *Panax quinquefolius*, *Panax ginseng*, *Panax notoginseng*, *Eucommia ulmoides*, *Aralia elata*, *Centella asiatica*, *Astragalus membranaceus* and *Malus domestica*. The deduced amino acid sequence of *TwFPS2* had 78–88% identity to those FPSs from *Glycyrrhiza uralensis*, *Astragalus membranaceus*, *Hevea brasiliensis*, *Euphorbia pekinensis*, *Medicago sativa*, *Pyrus communis*, *Aquilaria sinensis*, *Gossypium hirsutum*, and *Gentiana lutea*. The two proteins are highly conserved; the sequence identity was 77.26%, and the amino acid identity was 80.12%. Both *TwFPS1* and *TwFPS2*had five conserved regions, which were numbered I to V [27]. The highly conserved aspartate-rich motif in region II with the sequence DDXX(XX)D is called FARM (first Asp-rich motif), which is highly conserved in all known prenyltransferases and which has been designated as the chain length determination region [28]. Region V with the sequence DDXXD is called SARM (second Asp-rich motif). These regions marked with lines are characteristic of prenyltransferases that can be used to synthesise isoprenoid diphosphates [29] (Fig 2).

**Fig 3:**
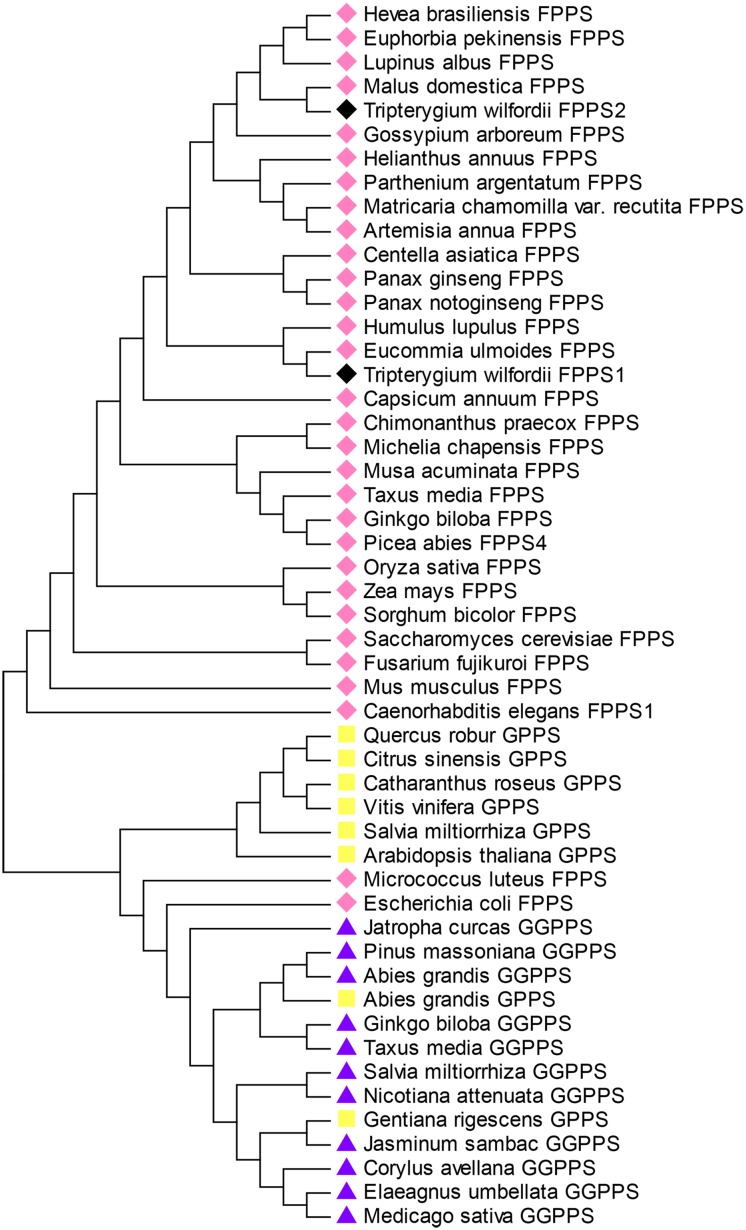
Phylogenetic tree of the amino acid sequences of isoprenyl diphosphate synthase of different organisms constructed by the neighbor-joining method on MEGA 5. GenBank accession numbers: *Hevea brasiliensis* (AY135188); *Euphorbia pekinensis* (ACN63187); *Lupinus albus* (P49351); *Malus domestica* (AAM08927); *Gossypium arboretum* (CAA72793); *Helianthus annuus* (AAC78557); *Parthenium argentatum* (CAA57892); *Matricaria chamomilla* var. recutita (ABS11699); *Artemisia annua* (AAD17204); *Centella asiatica* (AAV58896); *Panax ginseng* (AAY87903); *Panax notoginseng* (AAY53905); *Humulus lupulus* (BAB40665); *Eucommia ulmoides* (AB052681); *Capsicum annuum* (CAA59170); *Chimonanthus praecox* (ACJ38671); *Michelia chapensis* (GQ214406); *Musa acuminate* (AAL82595); *Taxus media* (AAS19931); *Ginkgo biloba* (AY389818); *Picea abies* (ACA21460); *Oryza sativa* (O04882); *Zea mays* (P49353.1); *Sorghum bicolor* (XP_002441458); *Saccharomyces cerevisiae* (p08524); *Fusarium fujikuroi* (CAA65641); *Mus musculus* (AAL09445); *Caenorhabditis elegans* (CAB03221); *Quercus robur* (CAC20852); *Citrus sinensis* (CAC16851); *Catharanthus roseus* (AHA82035); *Vitis vinifera* (AAR08151); *Salvia miltiorrhiza* (AEZ55677); *Arabidopsis thaliana* (NP_001031483); *Micrococcus luteus* (BAA25265); *Escherichia coli* (BAA00599); *Jatropha curcas* (ADD82422); *Pinus massoniana* (AGU43761); *Abies grandis* (AAL17614.2); *Abies grandis* (AAN01133); *Ginkgo biloba* (AAQ72786); *Taxus x media* (AAS67008); *Salvia miltiorrhiza* (ACJ66778); *Nicotiana attenuate* (ABQ53935); *Gentiana rigescens* (AHK06853); *Jasminum sambac* (AIY24421); *Corylus avellana* (ABW06960); *Elaeagnus umbellate* (ACO59905); *Medicago sativa* (ADG01841).

A phylogenetic tree of isoprenyl diphosphate synthases including GPPS, FPPS and GGPPS from different organisms was constructed to investigate the evolutionary relations. All of the plant isoprenyl diphosphate synthases sequences were separated into three main groups. *T*. *wilfordii1 and T*. *wilfordii2* clustered with 28 FPPS sequences. *A*. *grandis*, *G*. *rigescens* GPPS and *M*. *luteus*, *E*. *coli* FPPS clustered with 11 GGPPS sequences. This tree showed that FPSs evolved from a common ancestor and that the two *TwFPSs* belong to the clade of the plants (Fig 3). *T*. *wilfordii1* and *Eucommia ulmoides* were classified into one cluster, and *T*. *wilfordii 2* and *Malus domestica* were classified into one cluster. These clusters mean that these plants had the closest evolutionary relations. Moreover, these plants all belong to Angiospermae Dicotyledoneae. However, the two genes are not close, which suggests to us that a certain difference exists between *TwFPS1* and *TwFPS2*.

### Characterisation of *TwFPS1* and*TwFPS2* recombinant protein

**Fig 4:**
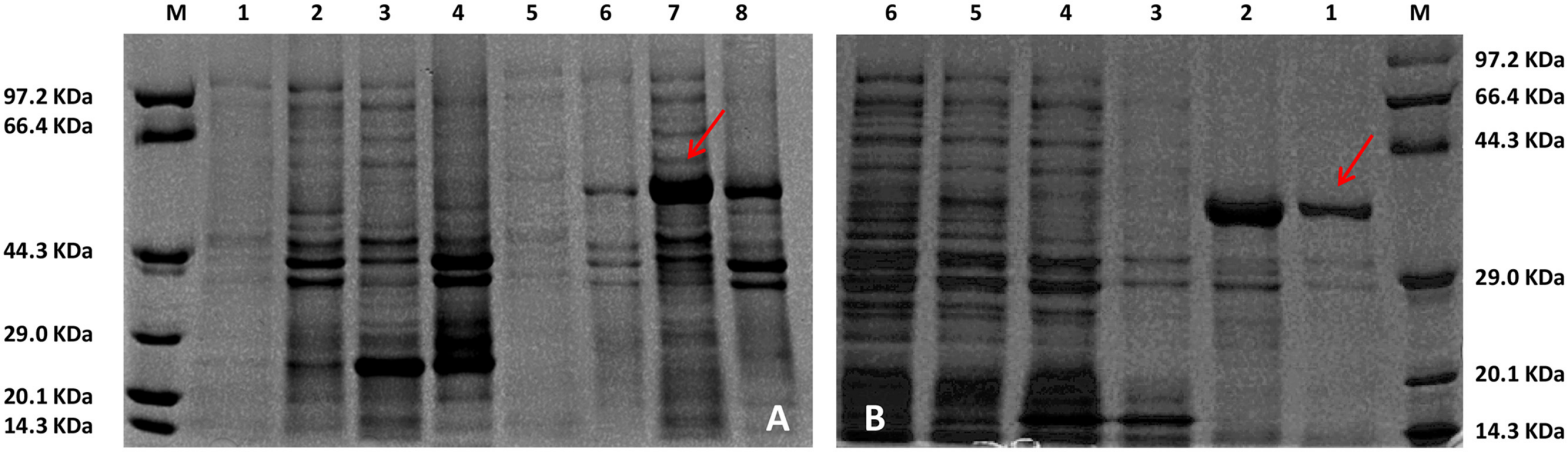
SDS-PAGE analysis of recombinant *TwFPS1*and *TwFPS2* protein expressed in *E*. *coli*. **A** Lane M, protein molecular weight marker(low); Lane 1, the supernatant of the empty vector without the induction; Lane2, the sediment of the empty vector without the induction; Lane 3, the supernatant of the empty vector with the induction; Lane 4, the sediment of the empty vector with the induction; Lane 5, the supernatant of the*TwFPS1* protein without the induction; Lane 6, the sediment of the *TwFPS1* protein without the induction; Lane7, the supernatant of the*TwFPS1* protein with the induction; Lane 8, the sediment of *TwFPS1* protein with the induction; **B** Lane M, protein molecular weight marker (low); Lane 1, the supernatant of the*TwFPS2*protein with the induction; Lane 2,the supernatant of the empty vector bacteria with the induction; Lane 3,the supernatant of the*TwFPS2* bacteria with the induction; Lane 4, the sediment of the empty vector with the induction; Lane 5, the supernatant of the empty vector with the induction; Lane 6, the sediment of *TwFPS2* protein with the induction.

The entire reading frame of *TwFPSs* was cloned into the pET-32a(+) vector and expressed in *E*. *coli* BL21 (DE3) cells to obtain the *TwFPS* protein for characterising the farnesylpyrophosphate synthase activity. After induction by IPTG, the recombinant protein was expressed. The molecular mass of *TwFPS1* (Fig 4A) fused with a Trx-tag and a His-tag on N-terminal is approximately 58 kDa, and the *TwFPS2* (Fig 4B) fusion protein is approximately 58 kDa, as determined by SDS-PAGE. The Trx-tag is a fusion tag and advantageous to the soluble protein expression. His-tag is a purification tag. Highly purified preparation of the protein can be obtained through purification with Ni-column affinity chromatography.

**Fig 5:**
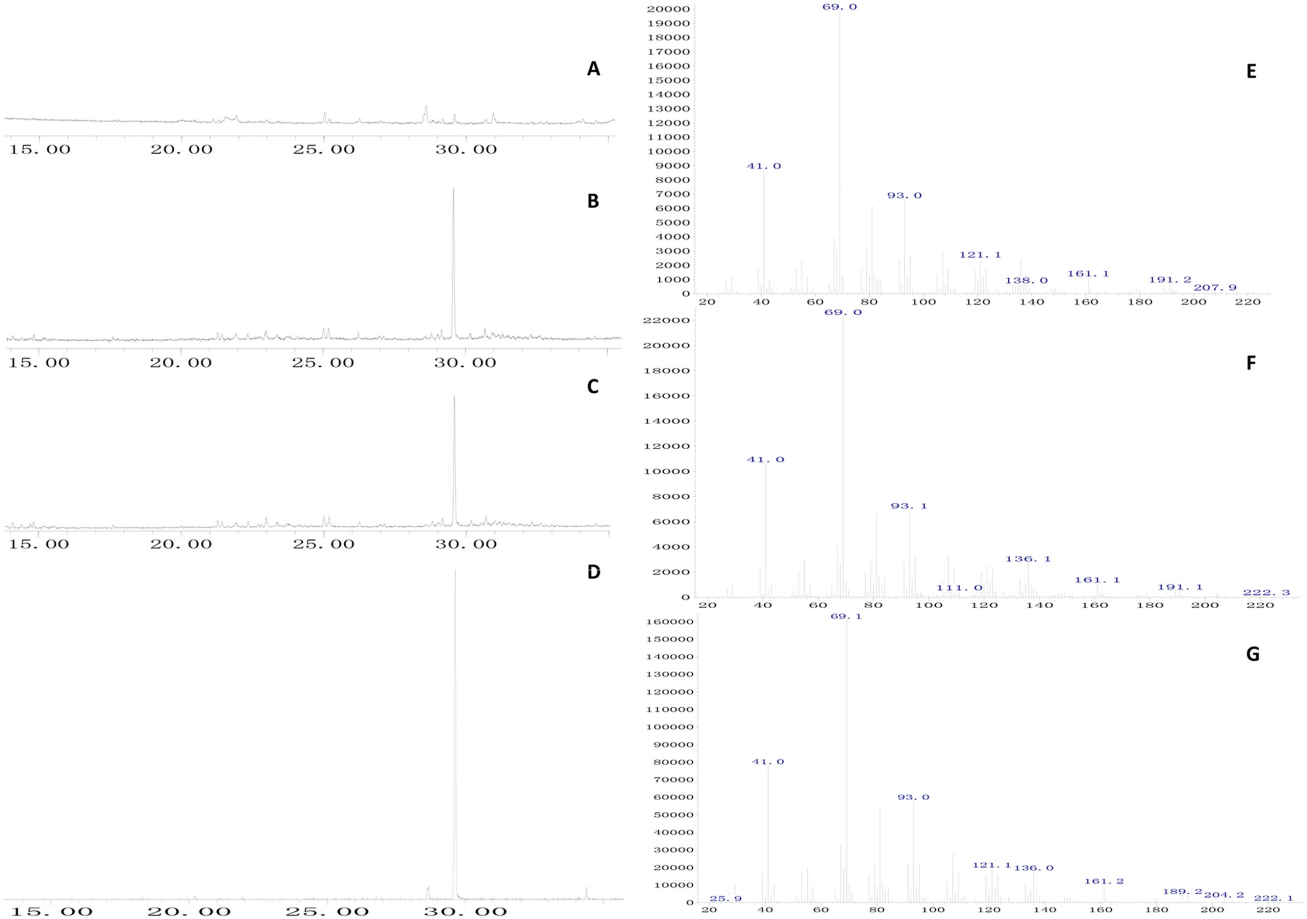
GC—MS analysis of reaction products catalyzed by purified recombinant *TwFPS* incubated with IPP and DMAPP. **A** Control (the empty vector). **B** The reaction products catalyzed by purified recombinant *TwFPS1* (IPP and DMAPP were added to the reaction mixture). **C** The reaction products catalyzed by purified recombinant *TwFPS2* (IPP and DMAPP were added to the reaction mixture). **D** GC—MS analysis of dephosphorylated FPP (farnesol) as standards. **E** The mass spectrogram of the reaction products catalyzed by purified recombinant *TwFPS1*.**F** The mass spectrogram of the reaction products catalyzed by purified recombinant *TwFPS2*. **G** The mass spectrogram of the dephosphorylated FPP(farnesol).

The purified proteins were assayed for farnesylpyrophosphate synthase catalytic activity. When the purified enzyme was incubated with DMAPP and IPP, the products had the same retention time as the farnesol standards (Fig 5A–5D). The GC retention time (RT) of farnesol was 29.588 min; *TwFPS1* samples of the product, 29.590 min; and *TwFPS2* samples of the product, 29.584 min. The blank control sample was not detected in the corresponding characteristic peak. Under GC-MS analysis (Fig 5E–5G), the FPS sample product qualified as farnesol, which had the characteristic peaks, including m/z = 222.0 (Molecular ion: M+) and m/z = 69.10 (CH3(CH3) = CHCH2-). Thus, these results indicated that the coding regions of *TwFPSs* encode functional FPP synthase.

### Tissue-specific and inducible expression of *TwFPS1* and*TwFPS2*

**Fig 6:**
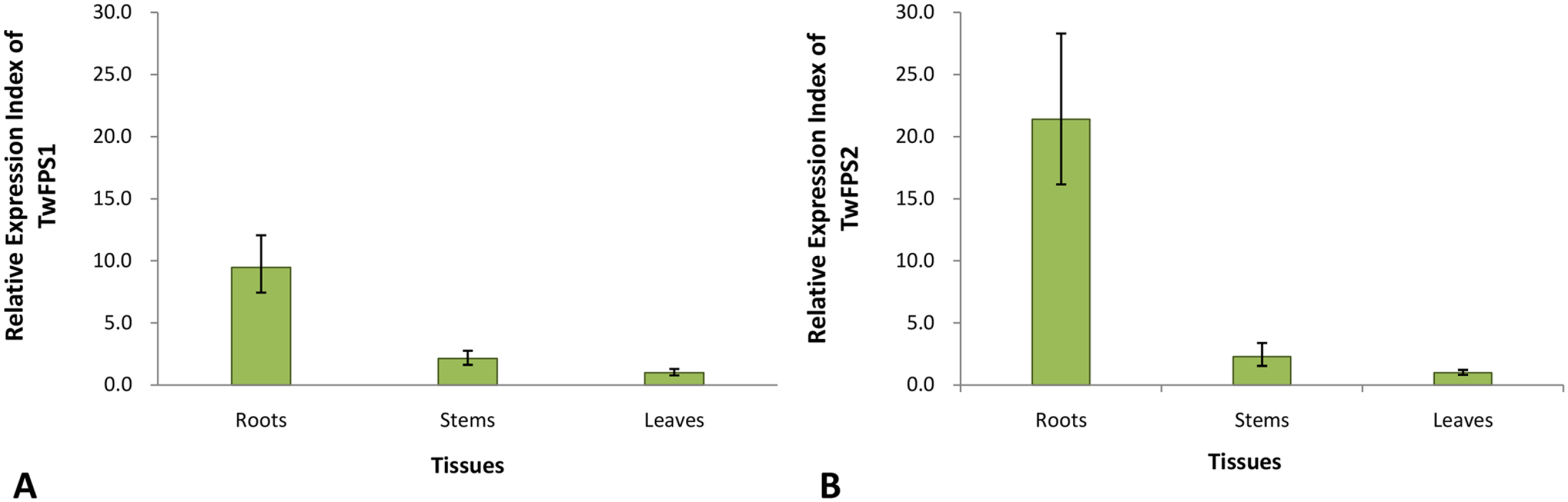
Expression patterns of *TwFPS1*and *TwFPS2* in different *T*. *wilfordii* tissues. Total RNA isolated from roots, stems and leaves. **A** *TwFPS1* expression in leaves was set as1; **B** *TwFPS2* expression in leaves was set as 1. Data are presented as mean±SE from three experimental replicates.

When total RNAs were isolated from the roots, stems and leaves of *T*. *wilfordii*, the lowest level of FPPS mRNA expression was found in leaves. Its value was set up as 1. The other FPPS expression tissues were evaluated relative to the leaves level. We found that *TwFPSs* are preferentially expressed in the roots. Among different tissues of *T*. *wilfordii*, the highest transcript levels of *TwFPS1* (Fig 6A) and *TwFPS2* (Fig 6B) were observed in the roots, and the lowest levels of *TwFPS1*and *TwFPS2*expression were found in the leaves. The highest levels of *TwFPS1* expression were observed in roots, about 9.5 fold higher than in leaves. The highest levels of *TwFPS2* expression were also observed in roots, about 21.4 fold higher than in leaves. It is indicated that roots are the main source of active contents.

**Fig 7:**
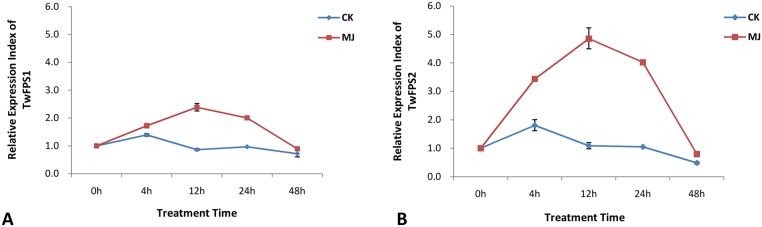
Expression profile of *TwFPS1* and *TwFPS2* when treated with 1mM methyl jasmonate (MeJA) over 48h. RT-PCR analysis was performed using total RNA isolated from suspension cells of *T*. *wilfordii*. **A** *TwFPS1* expression at 0h was set as 1; **B** *TwFPS2* expression at 0h was set as 1. Data are presented as mean±SE from three experimental replicates.

Moreover, real-time PCR analysis with cell suspensions at different developmental stages was also performed to examine the changes in the expression of *TwFPS* genes upon MeJA treatment. Specifically, MeJA caused a significant increase in *TwFPS* levels in *T*. *wilfordii* cell suspensions. Interestingly, the dynamic range of the induction varied. The levels of *TwFPS1*(Fig 7A) and *TwFPS2* (Fig 7B) expression both increased at first, peaked at 12 h, then decreased gradually, and the expression levels reached almost the same levels that of the control group after 48 h. The value of 0h was set up as 1 and the *TwFPSs* mRNA expression in other stage was evaluated relative to the 0h. The *TwFPS1* expression of MeJA group at 12h was 2.4-hold higher than control group. The *TwFPS2* expression of MeJA group at 12h was 4-hold higher than control group. These results indicated that the expression of *TwFPS2*relative to 0 h was higher than that of *TwFPS1*.

## Discussion

FPS plays a key role [30–32] in the catalytic reaction in isoprenoid biosynthesis; this step is considered rate-limiting. In the present study, we reported the molecular characterisation of two FPS genes from *T*. *wilfordii* for the first time. Knowledge regarding the regulation of sesquiterpene biosynthesis in medicinal plants is important. The results indicated that *T*. *wilfordii* contains a small FPS gene family that consists of at least two genes (*TwFPS1* and *TwFPS2*). These two genes shared a high level of sequence similarity in the coding region but not in noncoding regions. The root of *T*. *wilfordii* is the medicinal portion used in Chinese medicine. Pharmacological experiments confirmed that the primary active terpenoids of *T*. *wilfordii* showed strong pharmacological activities. The tissue expression analysis showed high *TwFPS* expression in the roots of *T*. *wilfordii*. This indicated that the biosynthesis of sesquiterpene compounds, such as wilfordine, occurs at roots. Moreover, the relative expression level of*TwFPS2* was higher than that of*TwFPS1*, suggesting that*TwFPS2*plays a leading role in terpene synthase biosynthesis in *T*. *wilfordii*. The analysis of FPS gene expression patterns in various plants demonstrated not only that the gene expression is tissue-specific [33–34] but also that the isoprenoid derivative contents increase [20, 30, 35–38]. The gene expression analysis showed that FPS genes could increase the isoprenoid substance contents in the plants [39–42]. In *Poria cocos*, there is a significant difference in total triterpenoids production between the control and experimental groups demonstrating that MeJA can potently stimulate triterpenoids biosynthesis [43]. The specific synthetic mechanism of terpene synthase biosynthesis requires further research.

In *Arabidopsis thaliana*, for example, FPS exists in the form of a small gene family. The FPS gene family encodes three isomers of FPS (FPS1S, FPS1L and FPS2). The primary difference between FPS1S and FPS1L is the N-terminus. FPS1S and FPS2 are found in plastids, and FPS1L is found in mitochondria [32]. The different metabolic channels determine their different functions and expression patterns. Thus, the secondary metabolism that FPS is involved in is complex, with great diversity and specificity. A study of rice shows that FPS exists in chloroplasts and in plastids [44]. FPS1 is expressed in different parts of the plants and participated in all life processes. FPS2 is primarily expressed in the flowers, as well as in the root tips of the lateral root and in the juncture with the primary root or secondary root [45]. The subcellular localisation prediction showed that the major localisation of *TwFPS1* is the cytoplasm; however, the localisation of *TwFPS2* is the cytoplasm or mitochondria. Therefore, a tentative inference of this result is that *TwFPS1* and *TwFPS2* participate in different metabolic channels.

These findings suggest that the expression of the native FPS genes in *T*. *wilfordii* result in the accumulation of the active components. The cloning and identification of key genes involved in the biosynthesis of active compounds from medicinal plants is important for the analysis of synthesis pathways. A recently established strategy in synthetic biology [46–49] is to use microorganisms such as *E*. *coli* to synthesize active ingredients found in medicinal plants, which will provide a new effective strategy and research approach for the sustainable utilisation of medicinal plant resources.

## Accession Number

The encoded nucleotide sequence can be found in the GenBank/NCBI data libraries. The number of *TwFPS1* is KM058711 and the number of *TwFPS2* is KM058712.
